# A Hospital for Negroes at Charleston, S. C.

**Published:** 1860-12

**Authors:** 


*A Hospital for Negroes* has been established at Charleston, S. C. The medical attendants are Dr. Cain, Physician, and Dr. Chisolm, Surgeon. It is to be opened for clinical teaching. —*Bos. Med. and Surg. Jour.*

